# Contrasting magma chemistry in the Candelaria IOCG district caused by changing tectonic regimes

**DOI:** 10.1038/s41598-024-61489-2

**Published:** 2024-05-11

**Authors:** R. Romero, F. Barra, M. Reich, A. Ojeda, M. J. Tapia, I. del Real, A. Simon

**Affiliations:** 1 Department of Geology and Millennium Nucleus for Metal Tracing Along Subduction, FCFM, Universidad de Chile, Plaza Ercilla 803, Santiago, Chile; 2 Instituto de Ciencias de la Tierra, Universidad Austral de Chile, Avenida Eduardo Morales Miranda, Edificio Emilio Pugín, Valdivia, Chile; 3 Department of Earth and Environmental Sciences, University of Michigan, Ann Arbor, MI USA

**Keywords:** Geochemistry, Geology, Petrology

## Abstract

Iron oxide-copper-gold (IOCG) deposits are a vital source of copper and critical elements for emerging clean technologies. Andean-type IOCG deposits form in continental arcs undergoing extension, and they have a temporal relationship with magmatism although they do not exhibit a close spatial relation with the causative intrusions. The processes required to form IOCG deposits and their potential connections to iron oxide–apatite (IOA)-type mineralization remain poorly constrained, as well as the characteristics of magmatism linked to both deposit types. Here we combine zircon U–Pb geochronology with zircon trace element geochemistry of intrusive rocks associated with the Candelaria deposit, one of the world’s largest IOCG deposits, to unravel distinctive signatures diagnostic of magmatic fertility. Our results reveal a marked transition in the geochemistry of intrusions in the Candelaria district, characterized by changes in the redox state, water content and temperature of magmas over time. The oldest magmatic stage (~ 128–125 Ma), prior to the formation of the Candelaria deposit, was characterized by zircon Eu/Eu* ratios of 0.20–0.42, and redox conditions of ΔFMQ − 0.4 to + 1.0. The earliest magmatic stage related to the formation of Fe-rich mineralization at Candelaria (118–115 Ma) exhibits low zircon Eu/Eu* ratios (0.09–0.18), low oxygen fugacity values (ΔFMQ ~− 1.8 to + 0.2) and relatively high crystallization temperatures. In contrast, the youngest stage at ~ 111–108 Ma shows higher zircon Eu/Eu* (~ 0.37–0.69), higher oxygen fugacity values (ΔFMQ ~  + 0.4 to + 1.3) and a decrease in crystallization temperatures, conditions that are favorable for the transport and precipitation of sulfur and chalcophile elements. We conclude that Candelaria was formed through two distinct ore-forming stages: the first associated with a reduced, high temperature, water-poor magma developed under a low tectonic stress, followed by a more oxidized, water-rich, and low temperature magmatic event related to a compressional regime. The first event led to Fe-rich and S-poor IOA-type mineralization, while the second event with geochemical signatures similar to those of porphyry copper systems, generated the Cu- and S-rich mineralization. This late stage overprinted preexisting IOA mineralization resulting in the formation of the giant Candelaria IOCG deposit.

## Introduction

In recent decades, there has been a growing interest in the exploration for mineral deposits that contain critical elements essential for the clean energy transition. Iron oxide-copper–gold (IOCG) deposits are an important source of copper but also of Au, P, F, Co, U and REEs^1–4^. In northern Chile, several IOCG, iron oxide-apatite (IOA) and porphyry Cu deposits (PCD) occur along the Coastal Cordillera metallogenic belt. These ore systems have a Jurassic–Cretaceous age and include two world-class IOCG deposits, namely Candelaria and Mantoverde^5–7^. The IOCG and IOA deposits are spatially associated with the Atacama Fault System (AFS) and dioritic to granodioritic intrusions. However, to date, the genetic relationship between these intrusions and the ore deposits has not been conclusively demonstrated.

Genetic models show that IOCG deposits are formed by hydrothermal fluids^1–4^, although the origin of these fluids has been debated. Several lines of evidence suggest a magmatic-hydrothermal origin for the fluids along with the influence of multiple fluid sources^3,8,9^. Specifically, studies on Andean-type IOCG deposits, which form in continental magmatic arcs under extensional conditions^4^, have shown that the ore-forming fluids are predominantly of a magmatic-hydrothermal nature, with minor but variable contributions from oxidized basinal brines^3,6,10,11^. Despite these advances, fundamental questions about the genesis of IOCG systems remain. One question relates to the similarities between IOA and IOCG deposit types, including the possibility that they might represent a continuum from deep Fe-rich mineralization to shallow Cu-rich ores, even though these mineralization styles are not necessarily synchronous^6,12,13^. Another fundamental question relates to the magmatic parameters of the coeval intrusions associated with IOCG and IOA-type mineralization, which may enhance the overall fertility. Hence, understanding the origin and geochemical characteristics of intrusions associated with Andean IOCG deposits is crucial for unraveling the genesis of these systems.

Zircon is a ubiquitous accessory phase in igneous rocks that has been extensively used for U–Pb geochronology. Furthermore, zircon can incorporate significant amounts of rare earth elements (REEs), making it a valuable tool for petrological and tectonic studies, particularly as a “fertility indicator” in the exploration for ore systems including porphyry Cu deposits (PCD)^14–19^. Several studies have investigated fertility indicators in PCDs; however, with the exception of a recent study on Precambrian IOCG systems in Australia^20^, to date no ore fertility studies have been undertaken in magmatic suites associated with IOCG deposits. Here, we present the first comprehensive study of trace element and U–Pb analyses in zircon from plutonic units of the Early Cretaceous Copiapó Batholith associated with the giant Candelaria IOCG deposit. Our main goal is to constrain first-order magmatic conditions that led to the formation of Candelaria, in particular the oxidation and hydration state of the melt, which can be possibly related to Fe and Cu mineralization. We further explore the geochemical signatures that can potentially serve as ore fertility indicators for the exploration of IOCG deposits in the Andean province, and potentially elsewhere.

## Geological setting

**Figure 1:**
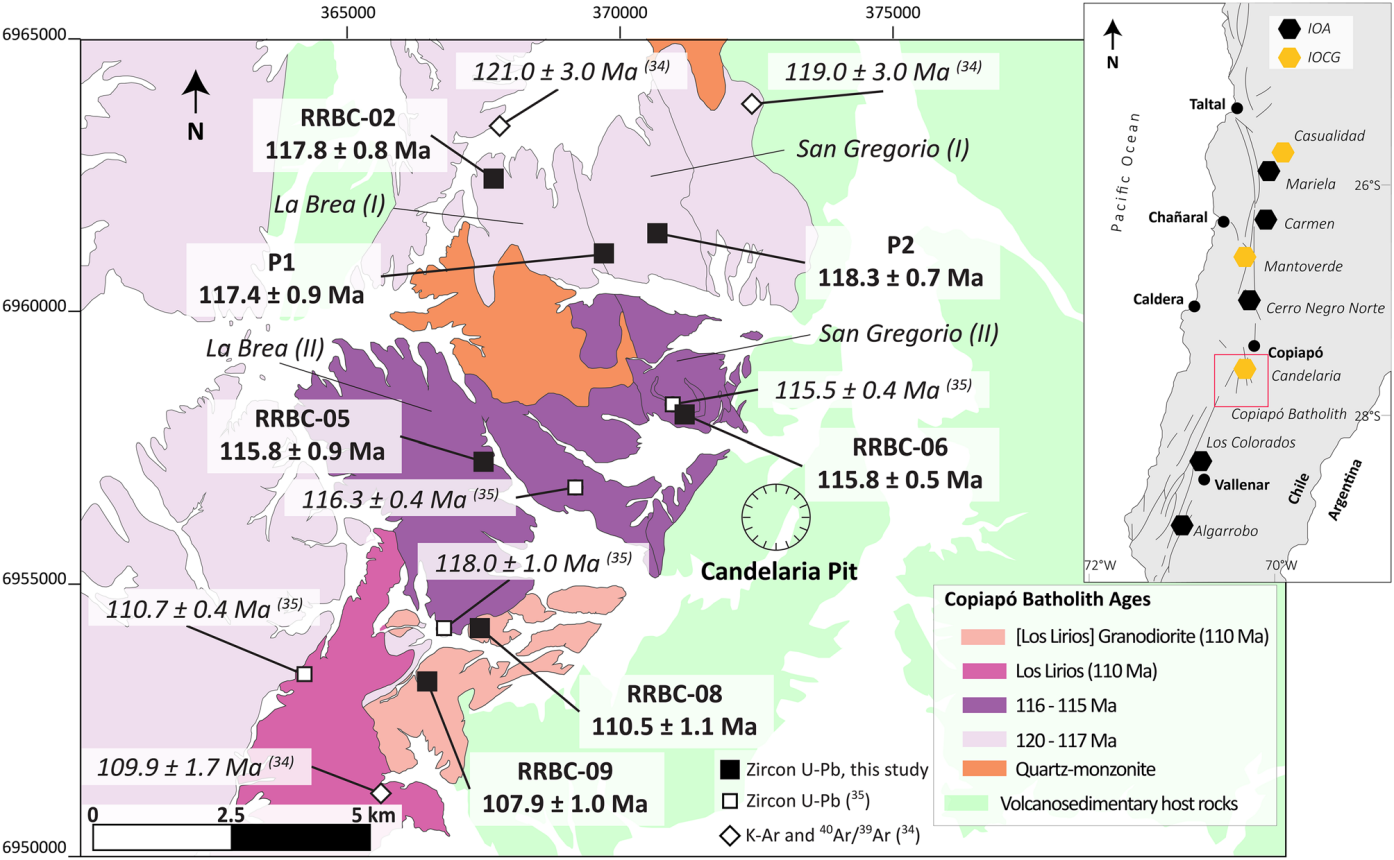
Geological map of the Candelaria district modified based on new geochronological data. Radiometric ages from this study and previous works^34,35^. Modified from del Real et al.^29^. IOA, iron oxide-apatite deposits; IOCG, iron oxide-copper–gold deposits.

The Coastal Cordillera of northern Chile extends between 21 and 33° S (Fig. 1), and hosts several types of ore deposits, including IOCG, IOA, stratabound Cu–Ag, and porphyry Cu systems^6,21–23^. These deposits are hosted mostly in Late Jurassic to Late Cretaceous volcanic and volcaniclastic rocks formed under an extensional tectonic regime with crustal thinning^5,24^.

Both the mineralization and plutonic activity in this period were strongly controlled by the Atacama Fault System (AFS)^25^, a structural system extending over 1000 km along the Coastal Cordillera of northern Chile. The AFS was characterized by an extensional regime between approximately 132–121 Ma, but it later transitioned into a sinistral strike-slip arc-parallel system. At ca. 112–110 Ma, the AFS become active under a compressional setting coinciding with the eastward migration of the magmatic arc^21,24,26,27^. During this period of crustal thickening, several porphyry Cu-Mo ± Au deposits such as Andacollo, Dos Amigos and Pajonales were emplaced between 116 and 90 Ma, forming a discontinuous belt to the east of the IOCG and IOA deposits^21–23^.

Situated within the Candelaria–Punta del Cobre district south of Copiapó in northern Chile, the Candelaria open-pit mine represents one of the world’s most important IOCG deposits^4,5^ with measured ore reserves of 478.2 Mt at 0.42% Cu^28^. In addition, underground mines in the district, including Candelaria Norte, Santos, and Alcaparrosa account for reserves of 270.7 Mt, with a Cu grade of 0.86%^28^. Other active mines in the district including Atacama Kozan (30 Mt at 1.5% Cu); Carola (60 Mt at 1.16% Cu), and Punta del Cobre (180 Mt at 0.9% Cu)^29^, collectively highlight the economic importance of this mining district.

In the Candelaria IOCG deposit, mineralization consists of magnetite, chalcopyrite, pyrite, and hematite^10,13,30,31^. The Cu-Fe mineralization occurs as breccias and stratiform bodies or mantos that are hosted in biotitized and actinolized andesitic to dacitic lavas and volcaniclastic units of the Cretaceous Punta del Cobre Formation^29^.

Chemical and isotopic analyses of magnetite and actinolite from drill cores in the Candelaria district reveal a vertical zonation. The S-poor deep levels are primarily associated with high-temperature processes^11,13^, while mineralization at shallow levels shows higher concentrations of Cu and Au^11^ related to lower temperatures and hydrothermal overgrowths^11,13^. These observations suggest a possible connection between IOA and IOCG-type mineralization.

**Figure 2:**
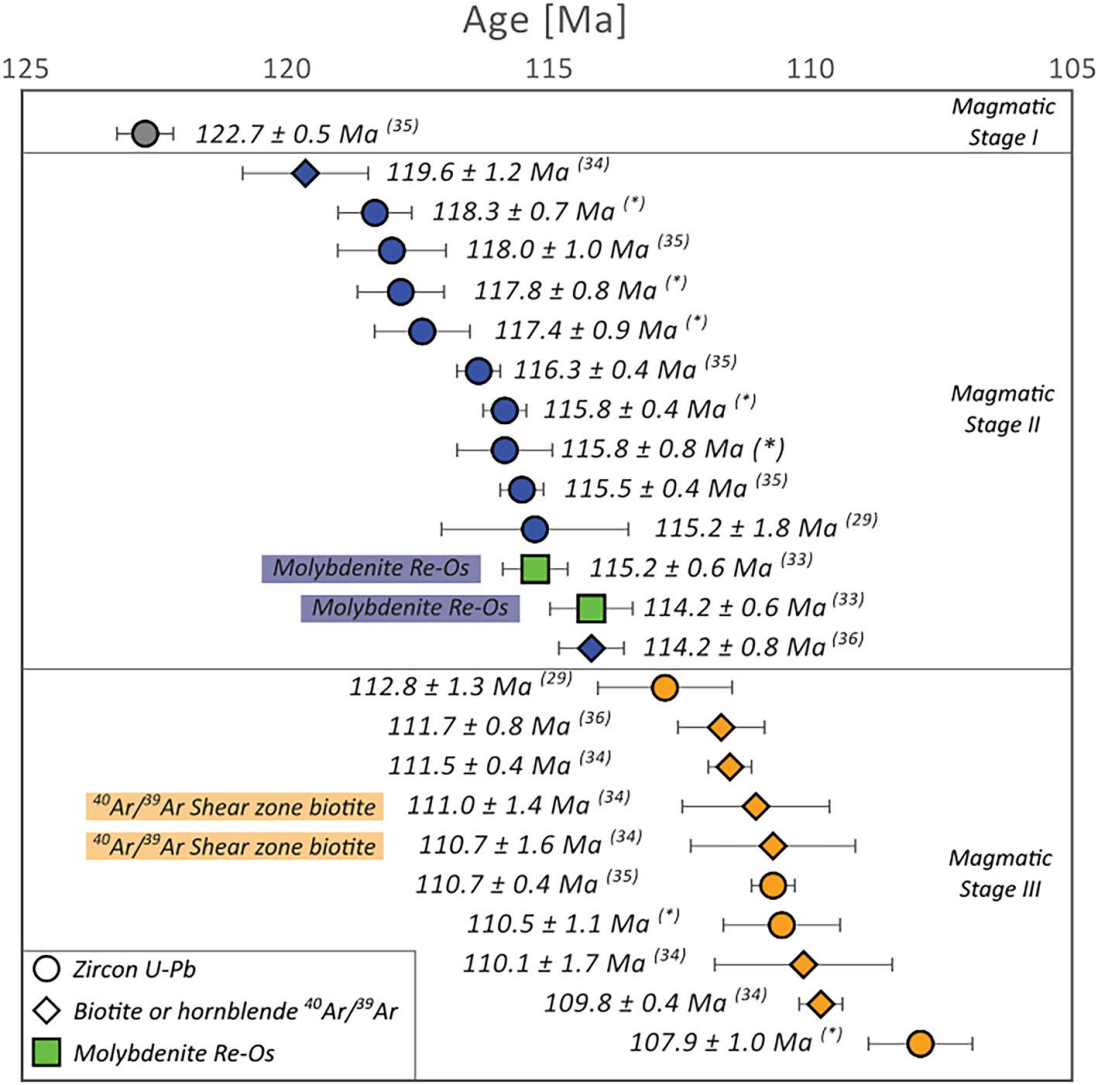
Compiled radiometric ages for the Candelaria district based on previously reported data^32–36^ and new U–Pb ages from this study^(*)^.

Four main intrusive units of the Copiapó Batholith outcrop near the Candelaria mine pit: La Brea (pyroxene–hornblende diorite), San Gregorio (amphibole monzodiorite to biotite monzogranite), Los Lirios (hornblende granodiorite to tonalite), and an unnamed granodiorite unit (Fig. 1). These intrusive units are classified as I-type, sub-alkaline to alkaline metaluminous granitoids^32^. Previously reported ages for these units in the Candelaria district range from 123 to 110 Ma^29,33–35^ (Fig. 2). Magmatism is nearly continuous between 118 and 115 Ma, followed by a magmatic lull extending for ca. 5 Myr. At 110 Ma, magmatic activity flares up resulting in the formation of the Los Lirios unit, located in the SW area of the district (Figs. 1 and 2).

The timing of Cu mineralization in the Candelaria deposit has been indirectly determined by using molybdenite Re–Os geochronology (115–114 Ma^33^), and also ^40^Ar/^39^Ar thermochronology on syntectonic biotite from the Candelaria shear zone (~ 111–110 Ma^34^), which is similar to the reported 111.7 ± 0.8 Ma ^40^Ar/^39^Ar age in amphibole related to the Cu mineralization^36^. These distinct ages have been interpreted to represent the timing of the Cu mineralization, and closely follow the aforementioned magmatic evolution. However, it should be noted that molybdenite is a scarce sulfide phase in Candelaria and hence, its precise paragenetic position has not been properly defined^30,31^. Furthermore, the reported Re–Os dates constrain the timing of molybdenite crystallization but not that of chalcopyrite or magnetite mineralization. Direct dating of Cu(–Fe) sulfides or iron oxides (magnetite) is a complex task, and usually yields radiometric ages with large uncertainties, which precludes constraining two (or more) temporally close events, for example, the magnetite-chalcopyrite Re–Os isochron for the Candelaria deposit (age = 110 ± 9 Ma, MSWD = 1.4^33^).

## Results and discussion

**Figure 3:**
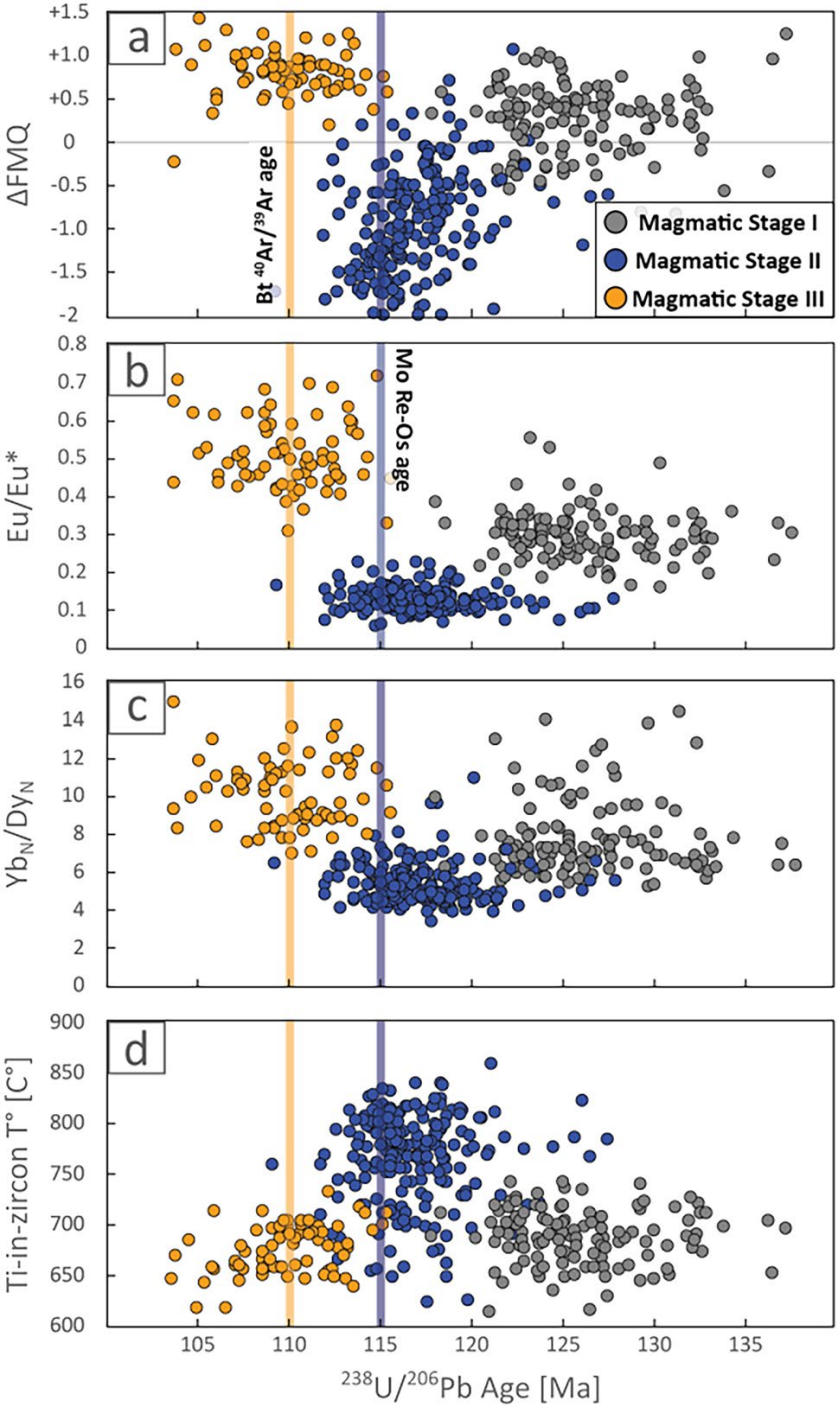
Zircon petrogenetic indicators of the Copiapó Batholith. Magmatic Stage I data are from Jara et al.^27^. Molybdenite (Mo) Re–Os^33^ and biotite (Bt) ^40^Ar/^39^Ar age^34^ are shown as constraints on the timing of mineralization in Candelaria.

Both zircon U–Pb dating and trace element analyses were conducted at the Mass Spectrometry Laboratory at the Department of Geology, Universidad de Chile (LEM–UChile), by using an Analyte G2 193 nm excimer laser ablation system coupled to an iCAP-Q quadrupole mass spectrometer. A detailed description of the methods employed can be found in the SM1 file. U–Pb ages and zircon trace element concentrations are summarized in Table 1 and reported in file SM2. Figure 3 presents zircon petrogenetic indicators of the intrusive units plotted against U–Pb ages used in the subsequent discussions. It also includes previously reported data for intrusive rocks located north of the study area, representing the magmatic conditions prior to the Copiapó Batholith^27^.

**Table 1:** Summary of weighted average ages along with mean and range (percentiles 5–95%) values for trace element parameters of the studied intrusions.

Sample	Unit	Description	Age [Ma]	Eu/Eu*	ΔFMQ	Ti-in-zircon T° [C°]	Yb_N_/Dy_N_	Stage
JJJD_01, 17, 18*	La Brea and Sierra Chicharra	Hornblende Granodiorites	128–125	0.30 [0.20–0.42]	+ 0.3 [− 0.4 to + 1]	688	7.8	I
RRBC-02	La Brea (I)	Hornblende Diorite	117.8 ± 0.8	0.12 [0.09–0.20]	− 0.4 [− 1.8 to + 0.7]	740	5.1	II
RRBC-05	La Brea (II)	Coarse grained Hornblende Monzodiorite	115.8 ± 0.9	0.14 [0.11–0.20]	− 1.5 [− 2 to − 0.6]	751	6.0	
P1	San Gregorio (I)	Biotite ± Hornblende Granodiorite	117.4 ± 0.9	0.13 [0.08–0.22]	− 0.7 [− 1.5 to − 0.2]	791	5.7	
P2	San Gregorio (I)	Biotite ± Hornblende Granodiorite	118.3 ± 0.7	0.14 [0.11–0.18]	− 0.5 [− 0.9 to 0.0]	802	4.8	
RRBC-06	San Gregorio (II)	Biotite Diorite, Bt + Ab alteration	115.8 ± 0.5	0.12 [0.07–0.16]	− 1.1 [− 1.7 to − 0.4]	766	5.2	
RRBC-08	Los Lirios Granodiorite	Hornblende Granodiorite	110.5 ± 1.1	0.48 [0.36–0.70]	+ 0.8 [+ 0.4 to + 1.4]	694	9.0	III
RRBC-09	Los Lirios Granodiorite	Hornblende Granodiorite	107.9 ± 1.0	0.54 [0.36–0.69]	+ 0.9 [+ 0.2 to + 1.3]	658	11.3	

*Data from Jara et al.^27^.

### U–Pb geochronology

The two samples from the La Brea pluton yielded ages of 117.8 ± 0.8 Ma and 115.8 ± 0.9 Ma. Similarly, two samples from the San Gregorio pluton have ages of 117.4 ± 0.9 and 118.3 ± 0.7 Ma (Table 1). However, a third sample from San Gregorio, collected near the Candelaria pit (sample RRBC-06), is considerably younger (115.8 ± 0.5 Ma; Table 1). Hence, two events are recorded in these intrusive units, an early event at ~ 118 Ma (La Brea I and San Gregorio I; Fig. 1) and a late event ca. 115 Ma (La Brea II and San Gregorio II; Fig. 1).

The samples from the unnamed granodiorite are the youngest in this study, with ages of 110.5 ± 1.1 Ma and 107.9 ± 1.0 Ma, which are similar to the reported 110.7 ± 0.4 Ma age for the Los Lirios pluton^35^. Hence, the former unit will be referred from here on as the Los Lirios Granodiorite suite.

Based on our data and previously published ages, we identified three main magmatic stages in the Candelaria district. The first phase, Magmatic Stage I (135–120 Ma), is represented by dacite dikes (125–123 Ma, zircon U–Pb^29^) and a barren granodiorite (~ 135 Ma, zircon U–Pb^29^). Coeval zircon trace element data from plutonic units located 10 km NW of the study area are included in the discussion as a part of Magmatic Stage I^27^ (Table 1, Fig. 3) to constrain the magmatic conditions prior to the formation of the Candelaria deposit.

Magmatic Stage II is represented by La Brea and San Gregorio with crystallization ages for the late event consistent with published molybdenite Re–Os ages, which were previously interpreted by Mathur et al.^33^ as the age of the Cu mineralization (115–114 Ma; Fig. 2). Magmatic Stage III, between 111 and 108 Ma, is represented by the Los Lirios Granodiorite suite. The age of this unit is concordant with ^40^Ar/^39^Ar ages obtained from syntectonic biotite from the Candelaria shear zone^34^ and amphibole associated with the Cu mineralization^36^.

### Magmatic evolution of the Candelaria district

Figure 3 shows the zircon petrogenetic indicators of the magmatic units plotted against U–Pb ages. Magmatic Stage I (128–125 Ma), prior to the formation of the Candelaria deposit, was characterized by redox conditions of ΔFMQ between − 0.4 and + 1.0 (mean ~  + 0.3) using the trace element in zircon oxybarometer^19^ (Fig. 3a), with evidence of early plagioclase fractionation based on the pronounced Eu anomalies in the REE pattern (Fig. 3b). The moderate fractionation of MREE, reflected in the Yb_N_/Dy_N_ ratio, may be attributed to early crystallization of small volumes of hornblende^18^ (Fig. 3c). These parameters—together with temperatures obtained by using the Ti-in-zircon thermometer^37^ (Fig. 3d)—point to geodynamic conditions typical of a convergent margin with moderate water contributions to the mantle wedge from the subducting slab, which would result in fractionation of high volumes of plagioclase and minor amphibole as an early hydrated phase.

Magmatic Stage II (118–115 Ma) reflects abrupt geochemical changes compared to previous magmatic conditions. The dominant feature of this stage is a significant decrease in oxygen fugacity, with values spanning between − 1.8 and + 0.2 (mean ΔFMQ ~ − 0.9), conditions which peak at ~ 115 Ma (Fig. 3a). In addition, the Eu anomaly becomes even more negative with Eu/Eu* values constrained in the lower 0.1–0.2 interval (mean ~ 0.1; Fig. 3b). Moreover, Fig. 3c displays a slight decrease in the zircon Yb_N_/Dy_N_ ratios ranging from ~ 6 to 4. These changes can be interpreted as representing a higher plagioclase fractionation occurring within a magmatic system at shallower depth, with higher temperature, and with lower water content, which would inhibit hornblende formation and reduce MREE fractionation. The decrease in the Eu/Eu* ratio during this period was primarily controlled by more plagioclase crystallization from less hydrous melts. Magmatic temperatures during this period increased significantly, reaching up to 850 °C, at ~ 115 Ma (Fig. 3d). Previous studies show that zircons crystallizing at lower temperatures preferentially incorporate smaller heavier REE, i.e., higher Yb_N_/Dy_N_ slope^38,39^. Hence, the lower Yb_N_/Dy_N_ ratios in zircons from Stage II can also be attributed to higher zircon crystallization temperatures (850 °C) due to a low water content in the melt.

Magmatic Stage III (111–108 Ma), exhibits a sharp increase in the magma redox state from approximately ΔFMQ + 0.4 to + 1.3 (mean ~  + 1). The Eu/Eu* ratio also increases reaching values between ~ 0.4 and 0.7 (mean 0.5), indicating early plagioclase suppression (Fig. 3a, b). Furthermore, these changes were coupled to a high involvement of amphibole in early stages of differentiation of the parental magma, based on MREE partitioning and a decrease in crystallization temperatures to values of 650–700 °C (Fig. 3c, d). These lower temperatures would contribute to a steeper REE pattern in zircon and consequently, a higher Yb_N_/Dy_N_ ratio^38^.

Therefore, we interpret that the timing of these changes, as identified in the trace element signatures in zircon from the Copiapó Batholith, closely follows the tectonic shifts along the AFS, where Magmatic Stage I corresponds to a period of normal subduction regime, Magmatic Stage II coincides with a transtensional setting, and Magmatic Stage III occurs during a more compressional regime starting at ~ 110 Ma.

The transition from Magmatic Stage I to Magmatic Stage II is interpreted to result from the shift from a presumably stress-neutral regime to the trench-parallel conditions identified along some segments of the AFS^21,24,26^. This configuration likely contributed to rapid magma ascent, shallow differentiation and short magmatic residence time, at least within this arc segment^40^.

The low tectonic stress condition during stage II effectively reduced the stability field of hornblende in this shallow level magma chamber and therefore increased the stability of Fe-Ti oxides^41^. This effect could explain the low zircon Yb_N_/Dy_N_ values due to lesser segregation of hornblende (and MREE) and the trend of reducing redox conditions caused by the preferential sequestration of Fe^+3^ over Fe^+2^^38,42^. Furthermore, the hydration state of magmas is closely related to prolonged stagnation at the crust-mantle boundary^43^. The low water content in these stage II magmas was probably caused by limited magma replenishments in shorter-lived, shallower magma storage chambers, hence less H_2_O accumulation in residual melts. Dissolved H_2_O has been identified as one of the primary regulating factors of oxygen fugacity (*f*O_2_) in melts due to its oxidizing nature^39^, thus contributing to the stage II low ΔFMQ values observed in this study.

On the other hand, the abrupt changes in the geochemical signature observed in zircons from Magmatic Stage III are coincident with a significant shift in plate motion. This shift resulted in the shallowing of the subducting plate, a transition to a compressional regime, and the eastward migration of the magmatic arc at ca. 112–110 Ma^21,26^. Consequently, it can be inferred that the thickening of the crust and subsequent entrapment of magmas at deeper crustal levels, i.e., higher pressures led to a prolonged, multi-cycle chamber replenishment fractionation history with higher water accumulation, which dramatically increased both the amphibole stability field and the oxygen fugacity of the melts during this stage, in contrast to the previous stage.

Although the changes in temperature, fractional crystallization processes and water content can be explained by changes in the tectonic stress regime at the continental margin, the significantly reduced redox state observed during Magmatic Stage II, particularly around 115 Ma invites discussion (Fig. 3). In particular, the distinct signatures of stages II and III closely align with the timing of molybdenite crystallization (115–114 Ma^33^) and the activity of the Candelaria fault zone around 110 Ma^34^, which were both previously interpreted as the age of Cu mineralization in Candelaria. This suggests a strong relationship between the magmatic processes during these stages and the formation of the IOCG mineralization in the Candelaria district. Hence, by linking the specific magmatic stages to the mineralization events, we can gain insights into the magmatic processes involved in the formation of Andean-type IOCG deposits.

### Implications for the formation of Andean IOCG deposits

**Figure 4:**
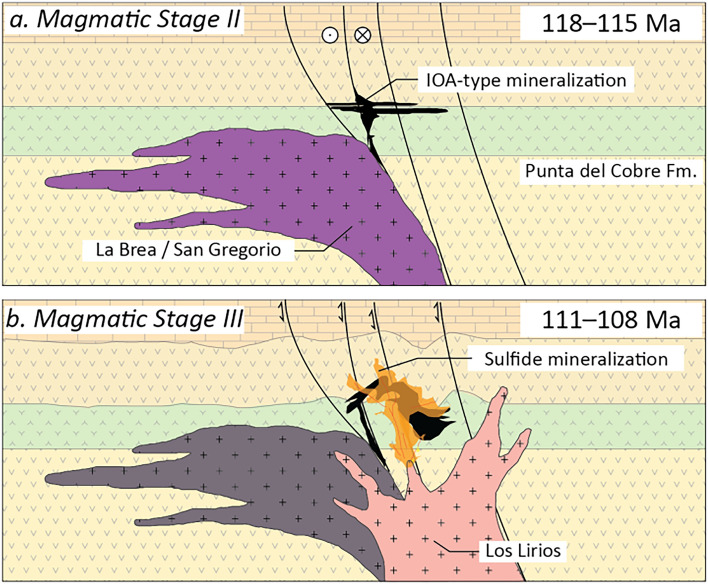
Proposed genetic model for the Candelaria deposit. Two main magmatic events were identified in Candelaria each related to a distinctive type of mineralization. (1) An early stage of IOA-type mineralization with massive magnetite segregation resulting in reduced magmatism, and (2) development of a sulfide-rich hydrothermal system derived from a water saturated oxidized magma that overprints pre-existing iron oxide ore bodies.

The magma chemistry variations, inferred from trace elements in zircon, can be correlated to different stages of the ore formation processes in the Candelaria district. Noteworthy is the unusually reducing conditions of Magmatic Stage II, which followed the emplacement of pre-mineralization intrusive units of Magmatic Stage I. Within the context of a typical continental arc setting, systems typically have ΔFMQ values between 0 and + 1^44,45^. Therefore, intrusions related to the early Fe-rich and S-poor IOA-type mineralization at the Candelaria deposit (Magmatic Stage II), characterized by ΔFMQ values ranging from − 1.8 to + 0.2, are clearly anomalous in Cordilleran arcs. The assimilation of highly reducing phases, such as graphite and organic matter-bearing rocks has been proposed as a mechanism for achieving magma reduction^46–48^. An alternative explanation involves sequestration of Fe^+3^ by magnetite crystallization. It has been recognized that magnetite formation and segregation decrease the Fe^+3^ content relative to Fe^+2^ during the evolution of an arc, which is invoked as a mechanism for sulfide (S^–2^) saturation and fractionation of chalcophile elements^42^. In a similar manner, we propose that formation of a large magnetite-bearing ore body during a narrow time interval at 115 Ma, could exacerbate this effect, resulting in the reduction of the residual magma of the Copiapó Batholith and representing the initial ore-forming stage of the Candelaria deposit (Fig. 4a).

During the Magmatic Stage III (111–108 Ma), more oxidized conditions (+ 0.4 to + 1.3) would allow more sulfur and metals (Cu, Au) to remain in residual melts due to less segregation of sulfides that sequester chalcophile elements^49–51^. In addition, the suppression of plagioclase during early amphibole crystallization has been mentioned as an indicator of significant magma hydration^16,17,44^. This process, coupled to lower magmatic temperatures, has been advocated as favorable for the formation of sulfide-rich porphyry Cu systems^16,17,51^. Therefore, similar conditions could be responsible for the Cu-rich event in Candelaria, which efficiently overprinted the early IOA-type mineralization stage (Fig. 4b).

The proposed two-stage model for the mineralization at Candelaria is consistent with the paragenetic sequence^11,30,31^, where an early iron oxide stage is followed by a sulfide stage characterized by the precipitation of abundant Cu sulfides, mainly chalcopyrite, which according to our data would have occurred ~ 5 Ma after the IOA-type mineralization stage. This superimposed sequence of events is further supported by: (1) the variability of magnetite trace elements concentrations interpreted as precipitation under high temperature conditions at deeper levels (IOA-type mineralization) to lower temperatures at shallower levels where the Cu sulfide ore forms (IOCG mineralization)^13^, (2) the two-stage variable chemistry of actinolite^11^, and (3) the Ni/Se ratios in pyrite, which are redox and temperature dependent^10^.

## Conclusions

The sharp variations in trace element patterns in zircon from the Copiapó Batholith likely responded to changing tectonic stress regimes in the continental margin during the Early Cretaceous, supporting a two-stage mineralization process for the formation of the world-class Candelaria deposit.

This model involves a high temperature, water-poor and reduced magma associated with an early IOA-type mineralization. This event reached its peak at 115 Ma and was followed by the main Cu sulfide stage, which may have occurred at ~ 110 Ma. This Cu sulfide stage was related to a magmatic system with low temperature, highly oxidized and hydrated conditions that enhanced the mobilization of metals (Cu, Au) and S from the magma. The Cu-rich magmatic-hydrothermal event overprinted the early IOA mineralization, resulting in the formation of the Fe oxide and Cu sulfide-rich deposit of Candelaria. This late event has similar magmatic characteristics as “fertile magmatism” in porphyry Cu systems, which suggests that the formation of Andean IOCG deposits may result from the superposition of magmatic-hydrothermal events of contrasting geochemical nature. These aspects need to be validated in detail in future studies, and contrasted with ore fertility data from other IOCG provinces worldwide.

Our results allow us to establish a characteristic signature of zircons associated with the formation of magnetite-rich bodies where the reducing conditions of the magma are diagnostic for exploration. The future challenge is to determine the triggers of magnetite crystallization and how consistent this signature is in other IOCG deposits.

### Supplementary Information

Supplementary Information 1.

Supplementary Information 2.

## Data Availability

The authors declare that all relevant data are available within the article and its Supplementary Information files.
