# Supplementary material for: Contrasting magma chemistry in the Candelaria IOCG district caused by changing tectonic regimes
Source: Sci Rep. 2024 May 11;14:10793. doi: 10.1038/s41598-024-61489-2 (PMC11088621; doi:10.1038/s41598-024-61489-2)
Supplement: Supplementary file 1 — Supplementary Information 1. [file 41598_2024_61489_MOESM1_ESM.doc]

**SUPPLEMENTARY MATERIAL**

**Simultaneous zircon U–Pb geochronology and trace element analysis.** Five to 10 kg of sample were processed for zircon mineral separation by electro-pulse disaggregation, Frantz magnetic barrier separation, and heavy liquid procedures at Zirchron LLCC (Tucson, Arizona). Approximately 100 zircon grains per sample were handpicked and mounted in epoxy resin. Plešovice (Slama et al., 2008) or SL2 (Gehrels et al., 2008) reference materials were used as a primary standard and 91500 (Wiedenbeck et al., 1995) as a secondary standard for Uranium-Lead dating. NIST 610 (USGS) was used as standard for trace element measurements. Polished mounts were analysed at the Mass Spectrometry Laboratory, University of Chile (LEM-UCHILE). Zircon crystal images (CL) were obtained by using a FEI Quanta 250 scanning electron microscope (SEM) coupled with a Centaurus sensor. Images were used to assess their internal structure. Simultaneous U–Pb geochronology and trace element concentrations were determined by laser ablation, inductively coupled plasma mass spectrometry (LA-ICP-MS). Analyses were carried out by using an Analyte G2 193 nm ArF excimer laser ablation system coupled to an iCAP-Q ICP-MS. Spot analyses (50 μm) were performed on the rims of the zircon grains. Each analysis considered 20 s of background followed by 50 s of data acquisition. The time-dependent drifts of U-Pb isotopic ratios and trace elements concentrations were corrected using a linear or exponential interpolation for every 5 and 15 unknown analyses, respectively. The reproducibility of the U–Pb geochronology was evaluated by comparison with the secondary reference material. Off-line selection and integration of signals, time-drift corrections and quantitative calibrations were performed using Iolite (Paton et al., 2011). Concordia diagrams and weighted mean calculations were constructed using Isoplot 4.0 (Ludwig, 2010). Uncertainties of individual analyses are quoted at the 2σ confidence level as propagated errors.

**Supplementary References**

Sláma, J., Košler, J., Condon, D.J., Crowley, J.L., Gerdes, A., Hanchar, J.M., Whitehouse, M.J., 2008. Plešovice zircon - a new natural reference material for U-Pb and Hf isotopic microanalysis. Chemical Geology, v. 249, p. 1-35.

Gehrels, G.E., Valencia, V., and Ruiz, J., 2008, Enhanced precision, accuracy, efficiency, and spatial resolution of U-Pb ages by laser ablation-multicollector-inductively coupled plasma-mass spectrometry. Geochemistry Geophysics Geosystems, v. 9, Q03017.

Wiedenbeck, M., Allé, P., Corfu, F., Griffin, W.L., Meier, M., Oberli, F., Quadt, A.V., Roddick, J.C., Spiegel, W., 1995. Three natural zircon standards for U-Th-Pb, Lu-Hf, trace element and REE analyses. Geostandards Newsletter, v. 19, p. 1-23.

Paton, C., Hellstrom, J., Paul, B., Woodhead, J., Hergt, J., 2011. Iolite: Freeware for the visualization and processing of mass spectrometric data. Journal of Analytical Atomic Spectrometry, v. 26, p. 2508-2518.

Ludwig, K., 2010. Isoplot/Ex version 4.1, a geochronological toolkit for Microsoft Excel. Berkeley Geochronology Center, Special Publication 4.
